# Improving OPC UA Publish-Subscribe Mechanism over UDP with Synchronization Algorithm and Multithreading Broker Application

**DOI:** 10.3390/s20195591

**Published:** 2020-09-29

**Authors:** Alexandru Ioana, Adrian Korodi

**Affiliations:** Department of Automation and Applied Informatics, Faculty of Automation and Computers, University Politehnica Timisoara, 300223 Timisoara, Romania; tm.alexandru@yahoo.com

**Keywords:** interoperability, industry 4.0, industrial protocols, OPC UA, publish-subscribe mechanism, time-synchronization, broker application, industrial internet of things

## Abstract

Communication protocols are evolving continuously as the interfacing and interoperability requirements are the foundation of Industry 4.0 and Industrial Internet of Things (IIoT), and the Open Platform Communication Unified Architecture (OPC UA) protocol is a major enabling technology. OPC UA was adopted by the industry, and research is continuously carried out to extend and to improve its capabilities, to fulfil the growing requirements of specific industries and hierarchical levels. Consistent issues that have to be approached are related to the latest specifications and the real-time context that could extend the applicability of the protocol and bring significant benefits in terms of speed, data volumes, footprint, security. The real-time context is essential in the automotive sector and it is highly developed within some specific protocols. The current work approaches first the conceptual analysis to improve the OPC UA interfacing using the Publish-Subscribe mechanism, focusing on real-time constraints and role distribution between entities, and considering some well-founded interfacing strategies from the automotive sector. The conceptual analysis is materialized into a solution that takes OPC UA Publish-Subscribe over User Datagram Protocol (UDP) mechanism to the next level by developing a synchronization algorithm and a multithreading broker application to obtain real time responsiveness and increased efficiency by lowering the publisher and the subscriber footprint and computational effort, reducing the difficulty of sending larger volumes of data for various subscribers and the charge on the network and services in terms of polling and filtering. The proof of concept is evaluated and the results prove the efficiency of the approach and the solution.

## 1. Introduction

Industry 4.0 and Industrial Internet of Things (IIoT) principles are guiding productivity, quality, efficiency, and safety, and at the same time are improving openness and competitiveness [1,2]. Structures, methods, objectives, and constraints are targeted to achieve improvements, and the outcome may be almost immediate as systems are able to communicate and understand each other [3,4], or may come after collecting data, identifying dependencies and patterns, defining and testing recipes, and reacting over the functioning systems [5,6]. All improvements are relying on interfacing, and a major enabling technology is the Open Platform Communication Unified Architecture (OPC UA) protocol [7,8].

OPC UA was first implemented on the Supervisory Control and Data Acquisition (SCADA) level in SCADA software environments and OPC UA centralizing servers, but the tendency was to use the advantages of the protocol and use it also on other levels. The technology entered using the Client-Server concept on the PLC level and companies slowly extended its functions to include as many characteristics and services as possible. Researches were carried out to extend and to improve OPC UA capabilities, to fulfil the growing requirements of specific industries and hierarchical levels. Studies were carried out trying to lower the application level to field devices [9,10]. Also, studies were tackling the cloud integration using OPC UA [11].

The first response to OPC UA issues that are ambitioning the academic and industrial communities, related to real-time, higher data volumes, footprint, came with the set of OPC UA specifications 14 [12], that relies on Publish-Subscribe mechanism. In work [13] the authors evaluate the applicability of OPC UA Publish-Subscribe in factory automation, while research [14] is setting the applicability prospect within the open62541 SDK [15], that is maintained and developed. Although effort have been undertaken by the research community, issues need to be clarified regarding the OPC UA Publish-Subscribe mechanism, especially related to the real-time context and to the architecture applicability and improvement for different industrial scenarios. With things being far from settled, studies are initiated to fasten the developments to fulfill requirements focusing on lower level protocols such as MQTT (e.g., OPC UA—MQTT gateway presented in [16], or combining Sparkplug B and MQTT in [17]).

Communication protocols that use the Publish-Subscribe paradigm are widely used in applications with real-time requirements. Even if the use cases might differ from industry to industry, besides different designs, particular implementations of the paradigm, and different protocols and standards used for accomplishing the mechanism, there are commonly adopted practices that proved decisive in assuring high performances in real-time applications in recent years. With the OPC UA’s Publish-Subscribe concept being still relatively recently defined, improvements and changes are expected in the near future, changes that should have foundational principles observed in legacy applications and in scenarios from multiple industries. The authors will refer to the OPC UA Publish-Subscribe mechanism in the current work only for describing the concepts specific to [12].

One of the communication protocols that implements the Publish-Subscribe mechanism in automotive is Scalable Service-Oriented Middleware over IP (SOME/IP). The Automotive Open System Architecture (AUTOSAR) standard describes certain requirements regarding the SOME/IP protocol for automotive application. In [18], the notification process between the Publisher and the Subscriber is mentioned by defining the mechanism responsible of informing the Subscriber about changed values or specific events occurrences, leaving also the possibility for the Subscriber to request changes of values or events or to verify status of variables and events through designated methods, allowing multiple strategies to be implemented from the architectural point of view. The standard splits responsibilities between the SOME/IP instance used for transporting changed values and the SOME/IP service discovery which is in charge of the subscribing and publishing processes. The notification process is described in term of possible strategies for different scenarios with the possibilities for cyclic notifications, on change notification, and conditional notification to be performed for the Subscriber offering a versatile path for implementing applications with specific Publish-Subscribe capabilities. The SOME/IP notifications message is also required to have certain particularities, worth mentioning being the sending of the length of the serialized payload, data that could prove useful for end-to-end verifications and filtering. On protocol level, besides the use of UDP, the SOME/IP protocol also uses TCP for handling network congestion, message lost, bit errors and other possible faults that may occur during transmissions. OPC UA’s Publish-Subscribe is focused on Subscribers constantly polling the network for messages, and subsequently filtering the received messages to identify if the desired information has been provided. With a dedicated notifying service or module, similarly to the design used in SOME/IP, OPC UA could extend the modularity of the Publish-Subscribe concept. Also, it could assure complete decoupling in term of Publishers and Subscribers as entities, and between responsibilities present in the system (who is sending the information, who is notifying the interested entities, who is responsible for security, who is managing the time base of the system, who is receiving the information). A set of dedicated services or modules that could be used as separate entities or as part of currently defined entities (Publisher, broker, Subscriber) is needed for assuring high quality of service and could represent necessary tools for the development of large scale application and robust architectures with high interaction capabilities between them. This middleware between the sending point and receiving point must assure all mechanisms that one scenario might need in an abstract way, making the scalability of the architecture accessible to all users based on their needs. In the case of the used transport protocol, OPC UA’s usage of UDP might not be sufficient in the future in the context of minimizing the fault tolerance of real time applications. The observable path is the combining of UDP and TCP at the transport layer for the highest level communication protocols that implement the Publish-Subscribe paradigm.

Besides the automotive field, implementation of Publish-Subscribe mechanism can be found in applications with real-time constraints and similar objectives in terms of connectivity, scalability and high performance. Data distribution services (DDS) are widely used in various fields like aeronautic, medical industry, power industry, etc. (e.g., [19,20]). Developed with real-time control objectives, DDS provides fault tolerant exchanges in real-time with low latencies and proficient filtering possibilities, assuring high modularity, a decoupled design of the Publish-Subscribe entities and time based operation possibilities allowing multiple synchronization strategies to be implemented increasing the robustness and scalability for a wide range of applications. Interaction between DDS and time sensitive networking (TSN) technology for increased time determinism possibilities is expected to be defined in a standardize way in quarter 4 of 2020. Being widely used, some of the practices applied by DDS may prove useful for OPC UA’s more recent concepts that target the real-time capabilities over a network. The adoption of TSN technology as standardized option should also be expected in the near future for OPC UA. However, before reaching that point, the design principles of the Publish-Subscribe mechanism might need to suffer changes. Particular services that could consolidate the binding between TSN and OPC UA, should be implemented as a prior step, enriching the current Publish-Subscribe concept and expanding the capabilities of the OPC UA standard in the context of IIoT. The key strategy for this scenario is to observe the needs and solutions for problems among multiple industries and establish goals for increasing the quality of service (QoS) on all possible areas.

The objectives of the current work are:

- To provide an analysis of the OPC UA interfacing using the Publish-Subscribe paradigm regarding real-time constraints and role distribution between entities, considering the current status of developments and some well-founded interfacing strategies from the automotive sector;
- To conceive and implement a solution regarding OPC UA Publish-Subscribe over UDP mechanism focused on a synchronization algorithm and a multithreading broker application that achieves real-time reactions and higher efficiency in order to extend the QoS and the applicability of the interfacing. The solution foresees to reduce the magnitude of the loosely coupled subscriber and publisher, the difficulty of sending larger volumes of data for various subscribers at high speeds, and the charge on the network and services in terms of polling and filtering. Availability and safety have to guide the approach, in that the solution must handle faults occurrence, focusing on fault detection, tolerance, and recovery.

The analysis of the OPC UA Publish-Subscribe mechanism focused on real-time constraints is presented in the second chapter, together with conceptual approaches regarding the proposed architecture and methodology. The third chapter details the proposed solution using a case study, including the algorithm for the synchronization mechanism, respectively describes and analyses the obtained results after the implementation and testing. The final chapter discusses the encountered challenges of the research and some direct applicability context.

## 2. Materials and Methods

The current chapter is focusing on in depth analysis of the OPC UA Publish-Subscribe concept with emphasis on design, real time requirements and possible implications towards ongoing approaches. An examination of the current mechanism and similar implementations on different communication protocols specific to different domains is provided with the purpose of identifying possible ways of improvement for future scenarios that might appear in the IIoT context. The last part addresses the synchronization algorithm, described in Section 3, alongside with a short description towards the necessity and objectives of the method. For the development of the current work, the SDK from [15] was used.

### 2.1. Publish-Subscribe Mechanism: Design and Architecture

The Publish-Subscribe mechanism is a pattern that relies on the exchange of information efficiently among 2 or more entities, with the basic principle that the Publisher will constantly publish an information or an event and that the Subscriber will be notified when the values have changed or when an event have occurred. The roles of the Publisher and Subscriber and all the between operations beside the sending and receiving the data can and it is recommended to be spread among more entities (nodes) in a system. The classical association between Server and Publisher roles and Client and Subscriber roles must be disregarded, and the focus must be towards what information is published, with the possibilities of more entities to publish the same information (e.g., a server and a backup server, or split topics among servers). Another focus should be towards the receiving of the information in the expected time interval, by the Subscribers. Having a more abstract view towards the system, the link between involved entities is less important than the link between information and target. The system should function with less information being taken into consideration (e.g., the number of subscribers of a publisher), and all the involved entities should spread the roles in the advantage of an efficient distribution of the information. In [21], the idea of an in-between mechanism is suggested, without any inter-object knowledge present in a system. This mechanism should take over some of the roles that can be performed without context regarding of the signification of the payload, but rather to serve as a middleware between Publisher entities and Subscriber entities, middleware that could provide significant advantage for large scale systems. One of the advantages mentioned by the author is that entities responsible with the transmission and receiving of the information can focus only on those topics, keeping low usage of their computational capabilities (this could translate to cheaper hardware for some nodes) and providing less usage of the network (this could translate to better response capabilities between participants inside the network). The message will be distributed in a more predictable manner, providing an abstract view regarding the relations between involved Publisher and Subscribers.

In [21], the problems of centralization regarding sensitive operation are discussed in complex systems based also on the content of the information. Besides the mentioning of different event types and subtypes for better subscribers’ manipulation, the single event servers are presented as disadvantages in systems that encapsulate many dependencies based in a single node of the system. Such entities that serve as publishers and also as event servers that make the decoupling of other Publishers and Subscribers possible, should be taken in consideration as high risks in any large scale system. For example, in the case of OPC UA, a factory that implements only one Publisher Server for interfacing with a cloud architecture, may suffer high losses if the server collapses and without backup responsibilities designated to another server. Without safety mechanism implemented for avoiding any critical impact in the system, important data can be lost and unknown amount of down time could be encountered alongside with possible total system failure if the server contains information regarding the binding of other entities (relations between publishers and subscribers). For having distributed responsibilities in the system, designated middleware entities must exist and be responsible for notifying subscribers when needed and also serve as the link entity between publisher and subscriber. Safety measures must also be implemented to monitor and react in case of failures from publishers and event servers. Only this way is the independence between the publisher concept and the subscriber concept guaranteed.

**Figure 1:**
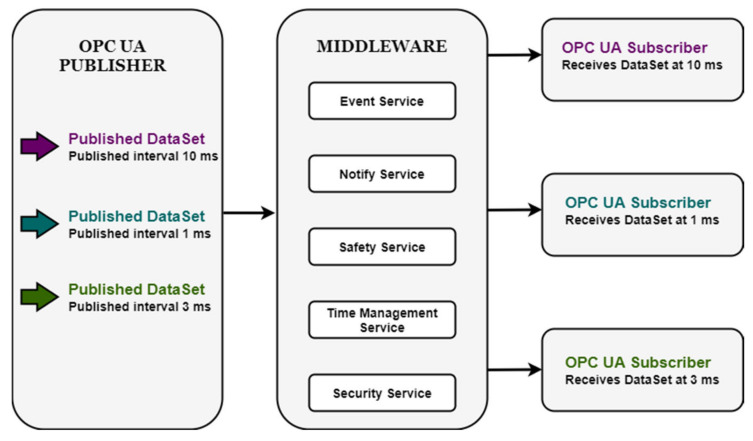
Hypothetical/Proposed Middleware Design with Services, in real time scenarios.

In [12], the roles of the publisher and subscribers are described as loosely coupled, with no impact in the information exchange between the number of existing Subscribers and a Publisher entity. The main relation between them is the common understanding of the specific DataSets involved and the messages publishing details. The message-oriented middleware is described as a connection to a multicast address in the case of the UDP messages and as a broker in the case of MQTT or AMQP messages. In the case where UDP is used at transport level, the publisher will be the one who send information to the multicast address. If the information is complex and it is targeted for different subscribers in different moments in time, without the notifying role well defined, subscribers will listen to any messages that comes to the middleware (multicast address), and will filter information based on the DataSetMetaData which also has to be transmitted by the same publisher to each subscriber before the point of filtering network messages. Document [12] refers to the DataSetMetaData as the information used at the Subscriber side for filtering the received network message, and options are described for obtaining the MetaData from the Publisher. One of the described methods is for the DataSetMetaData to be sent as a network message before the content of the DataSetMessages is changed. In the case of one Publisher to many Subscribers, this strategy could add complexity to the system and could create difficulties regarding time constraints between publishing and receiving the message. If security encryption is added to the context, a new entity called the security key server will be responsible with the administration of the security keys needed by the already involved entities. In a large robust system with many subscribers involved in relation with a publisher, assuming the encryption keys are received correctly by every subscribers, with them listening on all messages that are transmitted, decryption might be necessary as a first filter in the decoding of the message. Executing this step and other filtering operations until the subscriber will identify if the message is the expected one or not, can produce computational effort constantly with no immediate benefit on behalf of all subscribers. This factor will contribute to the achievement of real time requirements, and also in the case of multiple messages transmitted by the publisher in a dynamic way (assuming that also DataSetMetaData must be constantly sent) and decrypted, filtered and extracted by an unknown number of subscribers. The network itself could become incapable of managing to many exchanges in certain time intervals. The middleware as discussed in the context of an intermediate entity between a publisher and a subscriber, should hold information regarding the relations between publisher and subscribers, and in this case could also take over the distribution of the DataSetMetaData if necessary (see Figure 1).

Safety measures should be implemented for this kind of situations and constantly, the entity that contains the relations between publishers and subscribers, should verify the capabilities of the implied entities based on that information. In the ideal model, where the Publisher and Subscribers are decoupled from one another, the focus should be only on sending and receiving information. But especially for real time systems, safety mechanisms and guarantees regarding delivery time, decryption time and filtering time should be carefully taken in consideration, and could not be implemented without having the relation between all implied entities constantly stored in a node of the system. A possibility for such an entity in the OPC UA Pub-Sub mechanism could be represented by the future implementation of the The PubSub Directory.

Another strategy suggested in [21] details the notion of event service as the location where the relations between Publishers and Subscribers are located and describe a scenario where the Publisher is sharing a certain information or event type and from the event service receives the information regarding the interested subscribers. In this way the Publisher can inform directly the Subscribers about the events, however, this method is not as efficient as having an intermediary entity between a publisher and a subscriber, and by applying this strategy, the publisher and subscriber are not decoupled anymore. As the author mentioned, the approach of many-to-many should be taken in consideration when discussing the architecture of a robust and efficient system, where the principles of the Publish-Subscribe mechanism are intended to be implemented correctly.

In OPC UA, the filtering process of the network message on the Subscriber side creates a lot of steps from the receiving point of the message, until the content of the payload is accessible. These operations could imply more computational effort than necessary and could produce delays that could make difficult the achievement of real time requirements. The implementation of a Service somewhere at a level between the Publisher and Subscriber, that could do parts of the filtering of messages could help the Subscriber to not have to deal with the identity of the Publisher. The Subscriber could focus on the type of information that it targets and could probably provide some safety against man-in-the-middle attacks that could appear in the case of total independence among Publishers and Subscribers entities.

In the case where the middleware is represented by a broker in OPC UA, the design approach of the system regarding the decoupling of the Publishers and Subscribers is more close to the principles, but in this case the entity between publishers and subscribers is not linking applications on the same layer in the OSI model. The publisher indeed will connect to the broker and will be decoupled from the receivers of the information, the information will be received and stored by the broker but based on the transport protocol used by the publisher, the broker will be represented as a MQTT or AMQP receiving entity, so a lower layer receiver for a OPC UA publisher application. With the message being under this form (MQTT/AMQP format), the subscribers of the broker will be represented by other MQTT/AMQP applications, that will read the desired information without having to interact with layers above transport layer (where OPC UA is implemented) on the OSI model. So, the Publish/Subscribe mechanism is not fully implemented, rather an exchange between a publisher and a receiving entity (the broker) that is extracting the information and shares it with other applications that are MQTT/AMQP receivers. The use-case of this design is rather the interaction with Cloud architectures and with entities with different levels of understanding the OPC UA way of transmitting the information, the real time requirements in this case can be hard to implement. Safety features and services that could guarantee transmission in certain time intervals or message loss detection could prove to be more complex to implement in this type of design. From the security point of view, having a publisher with understanding of OPC UA and subscribers that are only MQTT/AMQP receivers, as stated in [12], the transport security between all entities (publisher, broker, subscribers) will be implemented. However, end-to-end security between Publisher and Subscriber, above transport layer and specific to OPC UA is not possible. In a classic case where controllers communicate with human–machine interfaces (HMIs) and where the decoupling of the entities is necessary, the usage of a design with a broker (MQTT/AMQP) based middleware could produce difficulties in assuring that the transmission is done in a fixed time cycle as it should happen. Even if the design is suited for many to many exchanges between devices with low cost and low complexity software, the assurance of the quality of service might prove more laborious than it should be.

In [22], a Publish/Subscribe mechanism is described in a design with a middleware (ROS middleware), where the publisher should not be aware about the subscriber’s identity or number. Every such detail will be managed by the middleware, providing an abstract view upon Publisher and Subscribers. This will allow the replacement of the publishers and the subscribers in real time if needed. Having this design, the middleware is implemented truly as a link between publisher and subscriber applications at the same level, with the complete same understanding regarding the technologies used. The implementation differs from the OPC UA broker middleware design (specific to MQTT/AMQP transport) where the endpoint applications may have different understanding of the OPC UA software.

In the automotive field, in the case of SOME/IP protocol, the Publish Subscribe pattern is responsible for the exchange of events between implied entities. The subscriber does not subscribe directly to a particular event but to an event group, similar to subscribing to a Published DataSet in the case of OPC UA, however, the subscription is done through the SOME/IP Service Discovery. The main Responsibility of the Service Discovery is to transmit the availability of services to any participants and to control the sending sequence of any event message. Having this service implemented allows for the Subscribers to receive only messages that are required. The approach of discovery is used also in other protocols that implement Publish/Subscribe mechanism. For example, DDS is using discovery of topics in a system not dependent on the application to have much focus on finding the data, but rather to receive it, improving in this way the decoupled design of the system and adding possibilities to implement safety mechanisms towards the detection of lost messages.

### 2.2. Time Synchronization in the Context of OPC UA and TSN Technology

In the context of TSN and OPC UA Publish Subscribe mechanism, the focus in the Industry Internet of Things is rapidly shifting towards real time functionality from the network level all the way to the application level. The objective of OPC UA Publish Subscribe is to make the next step in providing solutions for large scale applications that can exchange information in real time and that could be easily integrated in existing architectures. The TSN technology represents solutions for the data link layer to provide time accuracy in delivering messages inside a network using a series of standards for synchronizing clocks and defining time references for all the participants in the information exchange. With both technologies being developed incrementally towards the objective of fulfilling real time requirements, a new approach should be taken in consideration regarding the examination of other technologies and infrastructures with legacy in fulfilling real time requirements, within fields like automotive, aeronautics, military and other different industries. The way how the real time requirements are achieved and what impact has that on other topics like security, safety, quality of service, scalability and costs, must be highlighted. The comparison with the current context of the automation world in terms of needs and solutions should constantly improve the evolution of technology, understanding better what obstacles must be overcome in the upcoming, wide connected, world of IIOT.

The automotive field have been developing real time applications with different complexities in systems that interact at different levels inside a car. The AUTOSAR standard provides specific modules that are defining requirements regarding the time management and synchronization. One of the described modules is represented by the time synchronization module. Being part of both the classic and adaptive platform, the time synchronization implementation controls the time base used for synchronization between different nodes, and it can be described as an encapsulation of time protocols that makes possible the coordination between processes that run on different electronic control units (ECU’S) by establishing a common understanding of time. The adaptive version of the time synchronization is represented by a single module (SWS TimeSync), and includes also the synchronization provider (TSP) which for the classic platform is split in 3 modules, each one being protocol specific. Having in mind the objectives regarding the real time behavior for the OPC UA applications, it makes sense to analyze some of the requirements used for achieving real time behavior in AUTOSAR standard starting with time synchronization and also analyze the impact that such requirements have on different modules and technologies.

According to [23], the synchronization status should be available through interfaces for monitoring and detecting components of which behavior might be influenced by possible desynchronizations in receiving information. Another requirement refers to having a common understanding of time in al entities involved in the application, a possibility to share that time base over the ethernet and a synchronization based on the determined time base must be done between all Software Components and ECU’s involved for assuring accurate sensor-data processing from multiple sources. In the case of OPC UA for achieving hard real time synchronization the usage of TSN technology specific to the data link layer is necessary. One of the standards implemented by TSN is related to synchronizing the involved participants to one grandmaster clock as stated in [24]. The TSN implementation should guarantee some time delivery among network messages and should integrate the Publish-Subscribe mechanism of OPC UA in a real time context, targeting the controller to controller exchange. However, at the moment, it is hard to predict if the adoption of TSN technology will be enough for OPC UA large scale applications with real time requirements, using the current specifications of Publish-Subscribe mechanism. The monitoring of the synchronization between implied entities, in this case between OPC UA Publishers and Subscribers, is another important topic. Having in mind that, in large scale infrastructures, with multiple applications that will require different synchronization requirements, without dedicated interfaces that allow for the monitoring of each component involved, the management of the time bases and the interactions between real time applications would become overwhelming at some point. Dedicated services for time management and safety measures that could protect the user against desynchronization of operations, can improve the popularity and scalability of any communication protocol. This could represent a solution for real time applications, especially in the context of IIoT where the interaction not only between components but also between existing architectures will be necessary.

**Figure 2:**
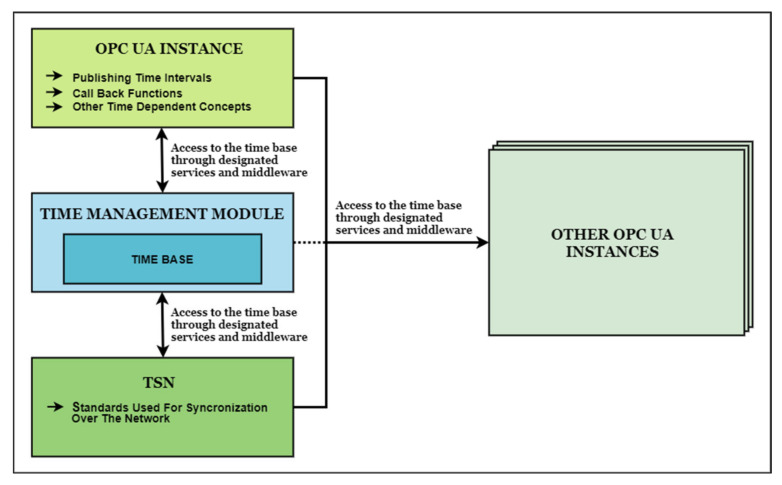
Proposed design with all entities relying to a common time base.

Other important requirements presented in [23] are mentioning notifications in case of elapsed time periods for operations that are expecting to receive information on certain time cycles for avoiding waiting states. The notifications of elapsed predetermined periods of time is presented in the document with the purpose of avoiding unnecessary polling and for synchronization over the network. The OPC UA similarities with this type of requirements are represented by the publishing time interval that can be predefined for the OPC UA Publisher. However, in the case of avoiding unnecessary polling on the network, the current Publish-Subscribe mechanism does not seem to exchange or share with dedicated services the publishing time intervals, so Subscribers are polling the network in the case of UDP messages transmitted by the Publisher. Future services that may increase the efficiency of Subscriber applications and decrease the computational effort necessary for such operation, by spreading the responsibilities to other entities (e.g., a notify service that will inform the subscriber when necessary, allowing the subscriber to only focus on receiving the information), could be developed with precise goals like preventing unnecessary polling. The time triggered transmissions are mentioned in the cases where further waiting of the next time cycle is no longer needed and operations can take place right then, allowing possible increases in efficiency in some cases and also allowing resynchronization if triggered transmissions are used. The transmission could be triggered at specific moments also in the case of OPC UA, however the triggering moment is specific to the application based on the hardware capabilities or operating system capabilities. Taking into consideration the time guarantees that the TSN could provide, the time references must be clearly defined between data link layer specific to TSN and OPC UA layers for assuring at first a synchronization of the operations inside the entity itself (publisher or subscriber) based on the same time base (see Figure 2). In the case of the AUTOSAR standard (and stated in the document), ways of accessing the time base must exist alongside with interfaces that provide information regarding desynchronization, time deviations, delays and other useful details from which the applications might benefit in a real case scenario. In any use case with strict time constraints, the defined time reference must be accessible. Any detail regarding the concept of time, even in the case of ethernet hardware clock or OS specific timers, could be used in synchronization processes internally and with other entities. Therefore, mechanisms that bind technologies that are time deterministic must exist preferably in a standardized way.

Current OPC UA time determinism is relying on the Publish-Subscribe concept and the TSN may provide enhanced capabilities for achieving real-time requirements. However, the perception of time, internally in entities involved in the information exchange over the network, and externally by different means and strategies of synchronization, must be developed further with the goal of standardization through well-defined services and middleware solutions as observed in other technologies that are real-time deterministic and which share similar goals. In the case of improving the QoS in every concept that implies time based operations, the notion of time itself should exist at the higher levels of the OSI model (OPC UA layers). Services and designated modules that manage and share access to the time base must be developed in concordance with future steps for elevating the performances and the possibilities of the applications, modules that might take in consideration the interaction with TSN technology that is also providing real time capabilities. From the publishing time intervals, to callback functions that are using a time base for the operations, the uniformity for the notion of time must be assured. Designated modules and services, alongside with the adoption of other useful standards and technologies for time synchronization and latency guarantees, are representing the next step in bringing OPC UA standard closer to overcoming the challenges present in the context of IIoT and Industry 4.0.

For achieving real time response between applications with different time bases, a synchronization is necessary. For the detailed case study in Section 3, where the broker application transmits information at a predetermined time interval, the goal is for the Subscriber to be synchronized with the Broker by being able to find the right moment to read the network message. A Synchronization Algorithm has been developed for this situation and it is described in detail in Section 3, alongside with other operations needed to identify only the desired message for each subscriber at any moment in time and to avoid the polling of the network as much as possible.

## 3. Case Study and Results

The OPC UA Publish-Subscribe mechanism in the context of real time requirements must be analyzed constantly for different scenarios present in the industry. With the rapid advancement of IIoT and the constant evolution and expansion of the technologies stacks involved in the process of information exchange among different points of interest, constant observation regarding any solutions and various comparisons with different needs and challenges specific to multiple domains, can produce significant improvements for the future development of large scale applications and for the identification of the best solution in any use case.

The authors are proposing the analysis of a multithreading broker application in the context of OPC UA Publish-Subscribe paradigm targeting UDP as transport protocol, with focus on real time constraints and role distribution among involved entities.

### 3.1. Architecture

**Figure 3:**
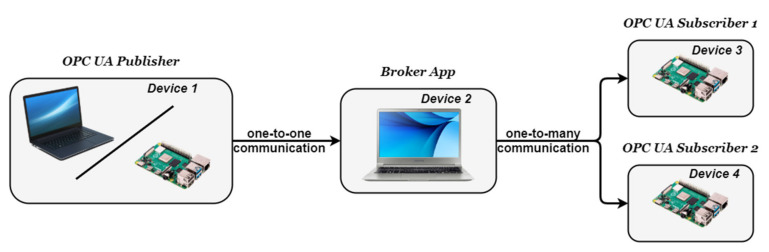
General Architecture of the Case Study.

The purpose of the broker application is to interface the Publisher and the Subscribers in an efficient manner, keeping the implementation close the specifications [12]. Having more entities present, the approach is to have each entity running on a separate device, with Linux-based OS as seen in Figure 3.

### 3.2. Case Study

For fast and efficient transmission, low computational effort and low resources usage, role distribution and synchronization regarding the involved entities, an application between the Publisher and the Subscriber can represent a feasible solution especially for use cases with real time constraints. Each entity involved in the case study has been conceived with certain objectives in place, and with different particularities towards fulfilling all the above-mentioned aspects.

#### 3.2.1. The OPC UA Publisher

The Publisher entity is responsible for delivering data without having knowledge about the final receivers and consumers of the information. As in [24], the current implementation follows the same sequence in terms of implementing the OPC UA Publish-Subscribe configuration components. The Publisher first has to be configured properly and initialize The Publish-Subscribe connection using the UA_Server_addPubSubConnection method. In this case, the Publisher aim to transmit information to a single entity, the Broker App, so the app will also have a connection initialized and configured to receive data only from the Publisher. The second step is the creation of the WritterGroup (see [12,24]) component, containing parameters responsible for creating the network message, one parameter being very important when taking in consideration real time constraints namely publishing Interval part of the UA_WriterGroupConfig data type. Targeting a one-to-one communication with the Subscriber part of the broker app, the intended transmitting data can be assembled in a more abstract manner, keeping simplicity in terms of implementation. It is expected that one of the purposes of having a Broker Application in this case to be represented by maintaining the Publisher application more abstract in terms of configuration components. Only necessary components will be configured with a basic configuration, and in terms of data encapsulation in designated, protocol specific structures. The payload is represented by a number in hexadecimal (e.g., 0xDC) without any knowledge regarding how the data will be split among subscribers, or how many target receivers there will be. As a hypothetical example, each 2 bits of the number may represent a value collected by a sensor, and the publisher is running on the field device that collects those data. The number will be represented by a DataSetMessage (see [12,24]) with only one DataSetMessage, field, keeping the structure of the payload as simple as it can be. The publishing time interval is set accordingly only to the needs of the Publisher entity, however, depending on the targeted scenario, the time interval should be set close to the data consumer needs. The last detail in the development of the publisher is the connection to the broker application. The publisher expects to share the information only with the broker, so it is not necessary to use a multicast address for the connection, the Ip of the device containing the broker app and an available port being enough to connect and publish the data.

#### 3.2.2. The Broker Application

In a design with a broker application, the improvement of the efficiency of the publish-subscribe mechanism is pursued. Besides filtering and routing only useful information to each subscriber, the increase of efficiency can be achieved by obtaining synchronization between involved entities. The current implementation follows all the above-mentioned aspects and aims to fulfill each objective in a manner close to the industry demands.

**Figure 4:**
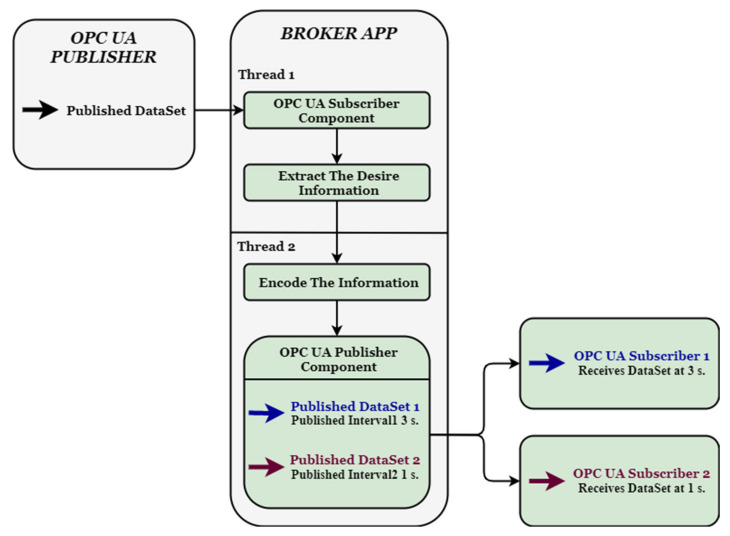
Architecture and Interaction of the Broker Application.

The Broker Application is formed by 2 different components, each one with different roles. The main roles are the receiving of the data from the OPC UA Publisher and the further publishing of extracted data at the desired time interval (to each subscriber). The main objectives of the Broker are to take over some of the roles involved in the Publish-Subscribe design, to achieve real time requirements by setting publishing intervals according to the needs of each subscriber and to execute most of the filtering at this specific point in the information exchange. Having to manage 2 different operations (receiving and transmitting further the data) with different time constraints, alongside with other specific tasks, a multithreading approach was chosen for a better performance and synchronization between the processes of the broker app. Although the [15] SDK does not provide multithreading, having to manage completely different operations made the approach possible for the current case. The high-level architecture of the broker app is split among 2 components, each one running on a different thread (see Figure 4).

The concept behind the Application is made with some assumptions in place for timing details, targeted information and unique ID, characteristics that should be provided from the subscriber side. Future iterations of the broker app might implement a third component responsible for a first exchange between the Broker and interested subscribers for such details, through a one shot transmission using the classic server-client paradigm, through a configuration step based on a Json config file at runtime, or through a periodic communication to a dedicated server that contains such details for the consumer entities. The above-mentioned details (timing details, targeted information and unique ID) are hard coded in the current version of the broker app for the present use case.

The first thread is dedicated to the Subscriber component. Being responsible of receiving the information from the Publisher, this component will also execute the extraction of desired data in specific target variables, based on preferences of each subscriber. The first step in the execution of the Broker Application is to initialize a PubSubConnection with the main OPC UA Publisher on a unicast address (one to one communication). After the connection is initialized, the component is starting to listen on the network for messages of interest. The implementation of the Subscribe process is done in a classic way, by interrogating everything that comes through the network with a recurrence as minimal as possible, to guarantee that none of the important DataSetMessages are lost, and by browsing through al all DatSetMessages fields and filtering data types.

The second thread is dedicated to the Publish component. Based on the number of subscribers and more on the time intervals on which the subscribers are expecting to receive the data, the component is initializing a OPC UA publisher instance, having multiple WritterGroups, each one with a different publishing time interval based on all subscribers needs. The data is stored after extraction and before being transmitted further to the subscribers, allowing historical data access if needed (data buffering is an essential feature regarding availability). Before the information is assigned to a DataSet, an encoding operation is necessary with a certain unique ID, particular to each subscriber. In the study case, the ID’s have been defined as a hex number (0xF for data of interest for subscriber1 and 0xA for data of interest for subscriber2). The encoding function is shifting the extracted information with 4 bits and assigns the ID on the first 4 less significant bits in the number obtained after the shifting. Therefore, the payload for the subscribers which as described in the OPC UA Publisher part is 0xDC becomes:

- xCF for subscriber1, where 0xC is the desired data for subscriber1 and 0xF the ID, and,
- xDA for subscriber2, where 0xD is the desired data for subscriber2 and 0xA the ID.

After the encoding is done, the new payloads are encapsulated in DataSetMessages and published on different time intervals by one of the WritterGroups. The transmission is done on a multicast address for common access among the subscribers (one-to-many communication) accordingly to [12].

#### 3.2.3. The OPC UA Subscribers

For the current study case, 2 subscriber entities were developed as consumers of the initial payload provided by the OPC UA Publisher, each one with different expectations in term of targeted data and real time behavior. For the exemplification of the current use case, the time intervals at which the subscribers are expecting information are 1 s for subscriber1 and 3 s for subscriber2. The concept is tested and functional also with time intervals under 10 milliseconds (at 3 milliseconds the behavior is similar as in the case of the targeted time intervals).

Each subscriber is implemented similarly, first steps being, in both applications, the initialization of the PubSubConnection and the establishment of the communication with the Broker App on the multicast address. Knowing that the broker will send only information of interest, there were avoided any unnecessary filtering operations specific to the OPC UA network message. The browse operation used usually for filtering through the content of the payload was also avoided, each subscriber knowing that the broker will send only the specific data and only at the desired time (in the ideal case). The decoding function based on ID is done for the ability of the Subscriber to classify arriving messages as Valid and Invalid for synchronization purposes discussed in Section 3.2.4.

The main objectives of having the broker app, were for the subscriber to avoid interrogating the network continuously for content and to use the publishing time interval of the Publisher concept for that, and to avoid as many filtering operations as possible by having the guarantee that only useful information will be received due to the synchronization. By achieving these objectives, the efficiency of the Subscriber application is increased, providing an advantage, especially if the targeted scenario is associating the subscribe application to a field device with low computational power and resources. Having in mind that the real time requirements are an important aspect of the OPC UA Publish-Subscribe concept, the implementation on the subscriber side followed the same aspect, with the instructions regarding the receiving of the information being executed only at time intervals equal to the expected time. For exemplification of the time between the calls of the receive instructions, the authors will use the term Delay in the next part of this chapter. The ideal state of functioning for the subscriber will be to have the Delay equal to the expected receiving time of the information. However, certain mechanisms were developed in the case of exiting the ideal case of functioning (desynchronization). All these mechanisms and more details are described in Section 3.2.4 regarding the synchronization algorithm developed for the concept design with the broker application.

#### 3.2.4. Synchronization Algorithm

With the broker application in place and with the subscribers expecting the correct messages at the correct time, for the best-case scenario, all configurations are correct. However, there are scenarios where only the right configuration of timing details for both the broker and the Subscribers is not enough for achieving real time synchronization between entities that do not have a common time base and do not exchange constantly time references through a notification mechanism. In these cases, the challenge is to synchronize separate entities in a dynamic way knowing that the approximative intervals of transmission and receive operations. For exemplification of the concept, the authors will use the term polling in the next part of this chapter for describing the desynchronization of the subscriber side from the broker app. The authors have defined the term polling based on 2 situations specific to the Subscriber’s state:

- The Subscriber receives information that the OPC UA receive function classifies as invalid data (not having the form of a network message or the operation haven’t been executed properly from network specific reasons), meaning that there is no way of knowing when the message will arrive and the re-execution of this function will eventually provide the desired message.
- The Subscriber receives a network message but, has a different type than the one desired and defined by the OPC UA protocol, again meaning that there is no way of knowing when the message of the desired type will arrive and the re-execution of the receive function will eventually provide the desired message.

Both situations specific to the defined polling state can be avoided only by obtaining synchronization between the send moment and the receive moment of the desired information.

**Figure 5:**
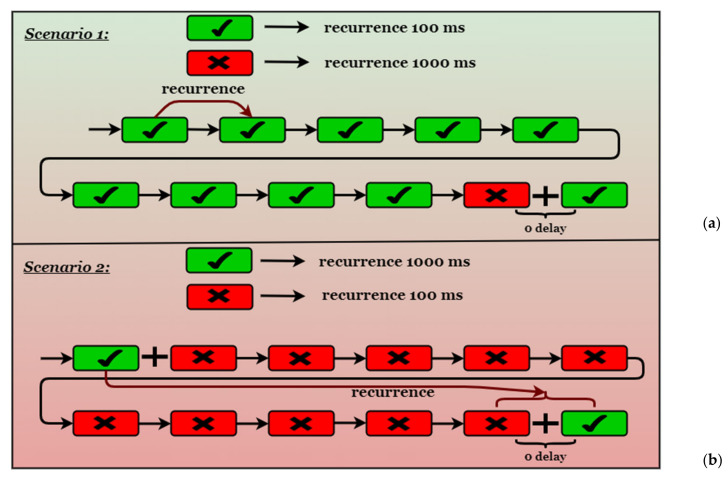
The 2 scenarios from the Subscriber Perspective regarding recurrence and validity of the messages. (**a**) Scenario 1–with high recurrence for valid messages. (**b**) Scenario 2–with low recurrence for valid messages.

From the configuration of the broker application to expediate messages designated to different subscribers based on an ID (see Section 3.2.2), and having two different delivery intervals for the messages, on a multicast address, from the Subscriber’s abstract perspective we can identify 2 different scenarios based on the validity of the messages (the subscriber will classify the message encoded with his ID as valid message and will classify the all other messages, despite the number of the involved subscribers as invalid messages), and on the recurrence of the messages (for exemplification purposes the recurrences chosen were 100 milliseconds for the valid messages and 1000 milliseconds for the invalid messages and vice versa) (see Figure 5).

In Scenario 1: If an invalid message has been received or a Polling state is detected, the delay becomes 0 (so the receive instructions are going to execute as fast as possible) until a synchronization event will occur (in the case of the algorithm, the synchronization event means the receiving of a valid message). If a synchronization event has occurred and a valid message has been received, the delay becomes again the desired receiving time interval specific to the Subscriber. From this point, there is assurance that the broker app sends that message using the same time interval, so it is safe not to execute the receiving operation until the next cycle and if the message has arrived, polling it is not necessary. From this point forward, if the network is stable, the Subscriber should be synchronized with the broker. The only case when desynchronization is possible, it is when the delivery time intervals for the 2 subscribers will intersect, and the broker will provide the messages very fast one after another, the subscriber not being sure which will be provided first. In this case, the receive instruction is executed and if the message that arrives first it is Invalid, the delay becomes again 0 and the instruction is repeated as fast as possible until the valid message is detected and not lost. Again, the apparition of the valid message is translated as a synchronization event and until the next intersection of delivering time intervals for the broker, the Subscriber is synchronized, and even if desynchronization appears, the mechanism of dynamically modifying the delay has the capacity to resynchronize the Subscriber with the broker. Between valid messages there will always be the desired recurrence, the polling is avoided from the moment the first sync is done (increasing efficiency), and the probability of receiving invalid messages is taken in consideration and the desynchronization is remediated as fast as possible. 

In Scenario 2: If an invalid message have been received or a Polling state is detected, the delay becomes 0 (so the receive instructions are going to execute as fast as possible) until a synchronization event will occur (in the case of the algorithm, the synchronization event means the receiving of a valid message). The behavior is the same as in Scenario 1 except that, at every cycle, a moment of intersection between delivering time intervals for the broker will occur, however, the mechanism of dynamically modifying the delay in the case of Invalid Messages will resync the broker and the Subscriber. Between valid messages there will always be the desired recurrence, the polling is avoided from the moment the first sync is done (increasing efficiency) and resync operation will take place at each cycle without producing lost messages.

In both scenarios, the implemented synchronization algorithm proves efficient and the testing of the implementation have confirmed that desired behavior is present on the broker application and on the Subscribers.

At this point, the purpose of encoding the information at the broker app side can truly be observed. In the adoption of a universal broker solution for UDP transmissions between OPC UA Publishers and Subscribers, the encoding process of the desired information can prove decisive for the synchronization process.

In the case when the broker application is shut down from any reasons, based on the synchronization algorithm, the subscribers will enter in a polling state and will execute the receive operation until a synchronization event will occur (in the case of the algorithm, the synchronization event means the receiving of a valid message), and after the synchronization will be made and if the network is stable, the polling state should not be reached again.

### 3.3. Results

The implementation of the broker application alongside with the Publisher and Subscribers entities was successfully realized, proving that the concept can be functional and that major goals have been achieved providing real advantages.

At the time of the implementation of the case study, the [15] SDK that was used did not provide a final version of the Subscriber API. From the authors point of view, the current solution for the receiving operation at the Subscriber’s side it is compliant to [12]. However, a final API being still in development, even if the authors did not identify any case, there are possibilities that at inferior software layers (beneath the software layer used for development of the Subscribe concept) to exist network interrogations or uses of buffers that stores all network messages that come through the multicast address, that might happen independently of the application layers of the SDK used by the authors. That is why the polling state was defined by the authors as explained in Section 3.2.4 at the application level specific to the use of the current SDK solutions. One major goal was the avoiding of the Polling state (avoiding the execution of more instructions than necessary more often than necessary, impossible without synchronization) so that the Subscriber to receive only messages of importance as an improvement of the current description of the receive concept as described in [12], “Subscribers shall be prepared to receive messages that they do not understand or are irrelevant. Each NetworkMessage provides unencrypted data in the NetworkMessage header to support identifying and filtering of relevant Publishers, DataSetMessages, DataSetClasses or other relevant message content”, and as implemented by [15] in compliance with [12] in the classic scenario where the broker application and synchronization algorithm does not exist as part of the Publish-Subscribe over UDP concept.

After the implementation phase, a series of result can be observed towards the concept from different perspectives:

1. The Publisher is implemented in a more abstract way than in the usual case, transmitting information for 2 subscribers with different preferences and time expectation by only using a basic configuration through a single Published DataSet. This can be seen as an advantage for transmitting larger amounts of data with less effort.
2. The broker application is using multithreading, so it is fast and the 2 components are working independent. Viable for operations with high complexity in cases of large scale applications.
3. The broker application is storing amounts of data that are passing form one side to the other. Data Buffering can be of interest is many cases with the OPC UA protocol.
4. The broker app can publish the information (last viable stored data) if needed, even in the case were the Publisher shuts down, serving as a backup server. In this way, some of the roles of the entities involved are taken over by other entities (in this case the publishing role), making the system more prepared in cases of failure. Also, safety measures can be implemented on the broker app to inform the data consumers about a malfunction with the source of data.
5. The broker application is providing data at the desired time intervals and by using the encoding strategy and the synchronization algorithm, it makes sure that the data is provided by delivering it at a stable rate (not faster that it is needed) in an efficient way.
6. The Subscribers are totally decoupled from the source of the information, without knowing any details from the main Publisher. This improves the concept of the Publisher-Subscriber mechanism as described in [12], “Publishers and Subscribers are loosely coupled. They often will not even know each other. Their primary relation is the shared understanding of specific types of data (DataSets), the publish characteristics of messages that include these data, and the message oriented middleware”.
7. The synchronization algorithm allows for the Subscribers to avoid as much as it can be avoided the Polling state and the filtering operations of different types. This translates to increased efficiency and decreased computational effort and resource usage for the host devices for the Subscriber entities.
8. The synchronization algorithm assures the Subscribers that the information will pe present at the right moment in time, in normal cases avoiding the polling state, providing also resync possibilities. This gives importance to the way of how the information is published (at a certain point in time), so by synchronizing the broker app and the Subscriber, real time requirements are achieved, and the solution can be targeted for controller-to-controller scenarios.
9. The current implementation is different from the broker used for the AMQP and MQTT transport protocols described in [12], on the design with the broker APP all involved entities have a common understanding of the OPC UA data types, all links involved in the transmission chain being OPC UA applications.

**Figure 6:**
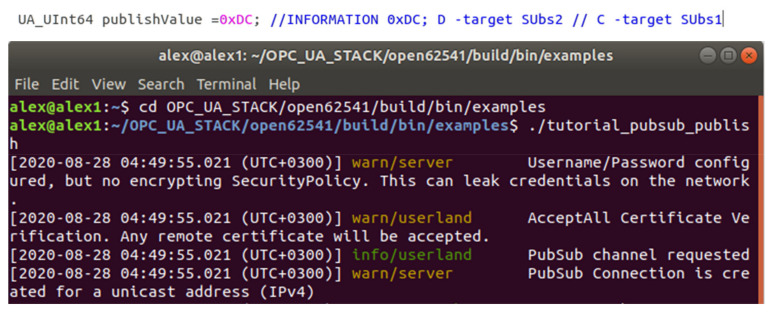
Terminal of the Publisher and the data that is sent to the broker.

**Figure 7:**
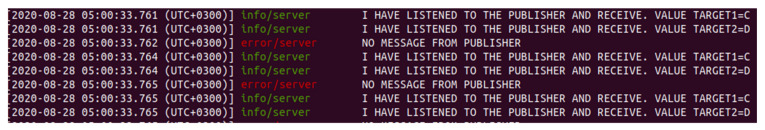
Terminal of the Broker App receiving data from the Publisher and transmitting it to the subscribers.

**Figure 8:**
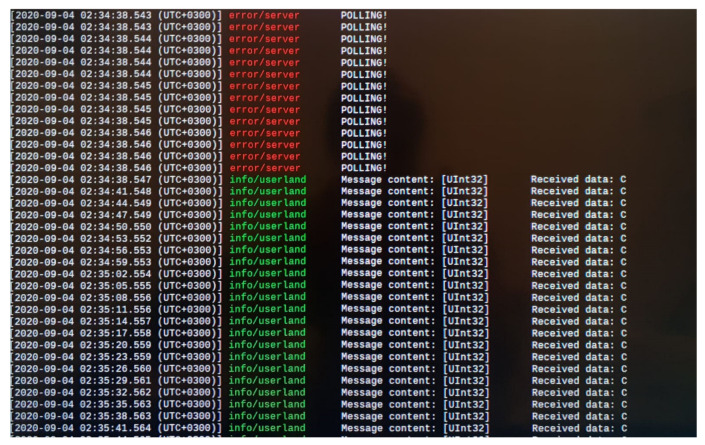
Terminal of Subscriber1 receiving the desired data at time intervals of 3 s.

**Figure 9:**
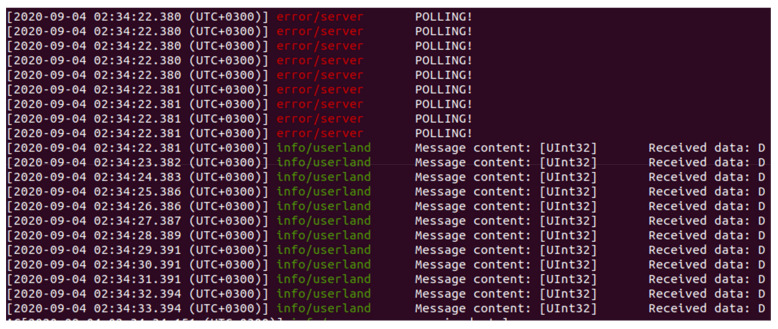
Terminal of Subscriber2 receiving the desired data at time intervals of 1 s.

Implementing the architecture described in Figure 3 and Figure 4, all the above-mentioned results can be observed in Figure 6, Figure 7, Figure 8 and Figure 9, each figure illustrating the terminal of an entity involved in the process, Publisher, Broker, Subscriber 1, and Subscriber 2.

An analysis of the results in terms of advantages and disadvantages from the development perspective is exposed in Table 1.

## 4. Discussion and Conclusions

The following paragraphs are discussing the challenges of the current research and some direct applicability context. 

A first obstacle in the implementation phase of the broker application was to identify what are the strengths and the weaknesses of the current design, and how can the strengths be applied further improving the behavior towards real time reactivity and increased efficiency of the Publisher and Subscriber entities.

One of the major challenges of the current work have been the identification of the synchronization mechanism for the subscriber’s side with the broker app. For achieving this objective, many different scenarios have been taken in consideration regarding what are the different possible states of the Subscriber at all moments in time, under what condition should the subscriber start to decrease and increase the delay time, how can the loss of messages be prevented even if the broker app and the Subscriber does not share the same time base. Another big challenge was represented by finding an encoding and decoding method that could be fast and simple for saving effort and time on both the broker app and on the Subscriber, and that could offer a clear way of distinguishing the desired message in the moments of time when the publisher component of the broker app will send multiple messages. The Subscriber was intended to be kept as simple as possible, an entity that just receives and consumes relevant network messages only at the right moment, so the filtering based on different subcomponents of the Publisher component was intended to be avoided, keeping a complete decoupled design, and keeping the functionality the same with less amount of information required from any entity involved.

Other challenges were determined by finding the right architecture for the broker app, for both of its components to work independently and efficiently and by implementing the Publisher and the Subscribers with a design as simple as possible, maintaining all the important operations on the broker’s side. The testing of the concept was difficult, having to use incremental versions of software for all the entities, the evolution of each version impacting how the others react. By having multiple devices involved, the testing of the results specific to different versions of each software has proven a difficult task and additional test code have been developed at different stages of the development phase.

The current conceptual approach has various direct applicability contexts. In the case of sensorial parametric controller that is exposing collected sensorial data, or the I/O controllers used to gather data from field devices, in order to communicate with PLCs or with redundant PLCs, the proposed concept would fit and advantage the OPC UA publish-subscribe mechanism on the edge of the network.

The synchronization and speed are essential in the case of cobots (collaborative robots) and in manufacturing industry they have to align to Industry 4.0 requirements in terms of protocol. From the authors industrial experience at various companies that are using robots, the challenges of cobots integration in the manufacturing process is to implement interfacing. Usually, inside cobots production cells, the best encountered functional development in the case of speed requirements was to eliminate any industrial interfacing and to implement simple strings-based interfacing in order to communicate with the local monitoring structure inside the cell. The flagship development [25] for universal robots could be improved with the current concept.

The manufacturing industry in general and also other industries could benefit by using the current concept. Considering an example of the end-of-line automotive manufacturing testing and packing, where a machine realizes automated optical inspection for ECU pins [26], after robotic pins insertion on boards, enclosure fixing and labeling, and before transporting and packing into boxes and final optical inspection of counting boards in boxes for sending to client companies. The automatic optical inspection (AOI) structure has to finalize the whole image processing in seconds and it has to communicate with the PLC on the same machine that fixes the enclosure, rotates the structure, and sends the ECU for packing or to part disposal. The first perspective would be that the optical inspection solution would have to send data to the PLC on the machine, to the upstream and downstream machines, to manufacturing execution systems (MES), for correct, coordinated, synchronized, safe, functioning of the involved processes. The presented solution would significantly contribute to a completely automated end-of-line manufacturing and testing, and would reduce the MES influence in the whole process functioning. 

The implementation of the broker application alongside with the synchronization algorithm displays the capabilities of the OPC UA protocol in use-cases with real time requirements and provides approaches that enhance the mechanism of OPC UA Publish-Subscribe towards increased efficiency and real time response for all entities involved in the information exchange process, expanding the area of development possibilities for applications covered by the IIoT context.

## Figures and Tables

**Table 1:** Case study results analysis.

Entity	Advantages	Disadvantages	Achievements
OPC UA Publishers	- moderate difficulty in implementation - easy configuration for different subscribers with different expectation - totally decoupled from the consumers of the information		- easy way of sending larger amounts of data for multiple subscribers with different expectation
Broker App	- multithreading capabilities - real time capabilities	- high complexity in implementation - an initial first step is needed for obtaining subscribers preferences and IDs (hard-coded information in the current implementation)	- real time behavior and synchronization with the subscribers - data buffering - backup publisher - safety capabilities in case the publisher is shutting down
OPC UA Subscribers	- easy/moderate difficulty in implementation - totally decoupled from the provider of the information - synchronization capabilities based on the described Synchronization Algorithm	- an initial first step is needed for transmitting preferences and ID (hard-coded information in the current implementation)	- real time behavior and synchronization with the Broker App - less polling of the network - less filtering for the desired information
